# Association between Genetically Proxied Inhibition of HMG-CoA Reductase and Age at Onset of Huntington’s Disease

**DOI:** 10.3390/brainsci12111551

**Published:** 2022-11-15

**Authors:** Yahui Zhu, Mao Li, Hongfen Wang, Fei Yang, Jiao Wang, Xusheng Huang

**Affiliations:** 1 Medical School of Chinese PLA, Beijing 100853, China; 2 Department of Neurology, First Medical Center, Chinese PLA General Hospital, Beijing 100853, China

**Keywords:** LDL cholesterol, HMG-CoA reductase inhibition, statins, Huntington’s disease, Mendelian randomization

## Abstract

Background: Previous studies have found that statins may play a potential role in the age at onset (AAO) of Huntington’s disease (HD). We performed this Mendelian randomization (MR) study to assess the association between genetically proxied inhibition of 3-hydroxy-3-methylglutaryl coenzyme A (HMG-CoA) reductase and low-density lipoprotein (LDL) cholesterol with age at onset of HD. Methods: Single-nucleotide polymorphisms (SNPs) in HMG-CoA reductase associated with LDL cholesterol in a genome-wide association study (GWAS) analysis were used. The summary data of residual AAO of HD were obtained from a GWAS meta-analysis (*n* = 9064 HD patients). MR estimates representing lifelong inhibition of drug targets were generated using random-effects inverse-variance weighted analysis. Results: Genetically proxied plasma LDL cholesterol (β = 0.039, 95% CI = −0.454 to 0.531) and HMG-CoA reductase inhibition equivalent to a 1 mmol/L (38.7 mg/dL) reduction in LDL cholesterol (β = −2.228, 95% CI = −4.830 to 0.374) were not associated with age at onset of HD. Conclusion: The plasma LDL cholesterol levels and the reduction of plasma LDL cholesterol levels by the inhibition of HMG-CoA reductase (i.e., statins) were not associated with the age of HD onset.

## 1. Introduction

Huntington’s disease (HD) is a neurodegenerative disease caused by the expansion of the cytosine-adenine-guanine (CAG) repeat in the huntingtin gene [1,2,3]. The main features of HD are cognitive, behavioral and motor deficits that gradually worsen over time. Although the expanded CAG repeat length explains approximately 60% of the individual variation in age at HD onset [4], other disease-modifying genes or environmental factors may also influence the age at onset (AAO) of HD [5,6]. Since HD is a fatal disease and there are currently no effective treatments, it is critical to identify interventions that can delay the onset of HD.

Hydroxy-3-methylglutaryl CoA (HMG-CoA) reductase inhibitors (statins), a class of low-density lipoprotein (LDL) cholesterol-lowering drugs, are commonly used to prevent and manage cardiovascular disease. HMG-CoA reductase inhibitors have neuroprotective effects in neurodegenerative diseases such as Alzheimer’s disease and Parkinson’s disease [7,8]. In animal models of HD, statins upregulate nitric oxide synthase, inhibit inflammation and reduce oxidative stress [9,10]. Studies of the premotor phase in HD patients showed that motor diagnosis was delayed in statin users than in non-users [11]. However, Aziz thought statin use would be a marker of hyperlipidemia, a better nutritional state which is good for HD patients [12]. The clinical relevance of these findings is unclear, as conventional observational analyses are susceptible to confounding factors and other biases.

Naturally occurring variations in the gene-encoding drug targets can be used for proxy regulation of these targets to examine the potential impact of their molecular inhibition on disease outcome (known as Mendelian randomization). Because genetic variation is almost randomly inherited at conception, analyses using variation as a proxy for targets intervention should be largely independent of confounders and not subject to reverse causality [13].

Since the effect of peripheral blood LDL-C levels and the use of statins to reduce LDL-C levels on the age of HD onset is unclear, we conducted the Mendelian randomization study. This is important to assess whether statins therapy is appropriate for HD patients. Thus, a Mendelian randomization analysis was used to examine the association of the drug target of statins (HMG-CoA reductase) with the age at onset of HD. In addition, we also examined the association of LDL cholesterol with age at onset of HD.

## 2. Materials and Methods

### 2.1. Study Design and Data Sources

We obtained genetic instruments of proxy HMG-CoA reductase and proxy LDL cholesterol levels from pooled data of LDL cholesterol levels GWAS meta-analysis in the Global Lipids Genetics Consortium [14].

At the genome-wide significance level (*p* <  5.0 × 10^−8^) and within the ±100 kb window of the HMGCR gene region, single nucleotide polymorphisms (SNPs) associated with LDL cholesterol were obtained. For the drug target, in order to increase the proportion of variance in the drug target explained by the instruments and to maximize the instruments’ strength, we allowed the SNPs used as proxies to be in weak disequilibrium (r^2^ < 0.20) with each other. Thus, five SNPs were generated as genetic instruments of proxy HMG-CoA reductase.

For proxy LDL cholesterol levels, 76 independent (r^2^ < 0.001) SNPs associated with LDL cholesterol at genome-wide significance levels were obtained, independent of the genomic position of variants.

Subsequently, in order to satisfy the hypothesis that instrumental variables (IVs) are not associated with confounding factors, we used the PhenoScanner_V2_ (http://www.phenoscanner.medschl.cam.ac.uk/) tool [15] to screen whether the selected SNPs were associated with potential confounding factors affecting the age of HD onset. When using the PhenoScanner tool, the genome-wide significance level was *p* < 5.0 × 10^−8^. Finally, the strength of each SNP using the F statistics (F = beta^2^/SE^2^) was evaluated [16]. To avoid weak instrumental variables, we excluded SNPs with less statistical strength (F statistics < 10). The proportion of variance was explained by each instrument, with SNP R^2^ calculated using the following formula [17]: R^2^ = 2β^2^EAF(1−EAF)/SD^2^, where EAF represents the effect allele frequency of the instrument SNP, and β denotes the effect size for SNP.

### 2.2. Outcomes

The primary outcome in this study was the residual AAO of HD defined by the Genetic Modifiers of Huntington’s Disease (GeM-HD) Consortium. The residual AAO of HD was calculated as the difference between the actually observed (diagnosis of clinical motor symptoms) age of onset and the expected (based on CAG repeat length) age of onset [18]. For example, a residual AAO of +5 indicated that the AAO of motor symptoms in premanifest HD mutation carriers was 5 years later than expected AAO. The GWAS meta-analysis of residual AAO included 9064 HD patients of European descent (4417 males and 4647 females).

### 2.3. Statistical Analysis

The MR method is based on three assumptions: (1) the genetic variants are associated with the exposure, (2) the IVs are not associated with confounding factors, and (3) the outcome is influenced only by the exposure, not by other pathways [19].

We applied different complementary methods (inverse-variance weighted (IVW), MR–Egger, weighted median and MR–PRESSO), which provided different hypotheses about horizontal pleiotropy. The IVW method, as the main method of this study, had the basic assumption of zero intercept and performed a weighted regression on the SNP–exposure effect and the SNP–outcome effect. When pleiotropy was present, the MR–Egger method provided more conservative estimates of causality and was less likely to produce exaggerated test statistics [20]. The weighted median approach assigned more weight to more precise instrumental variables, and estimates were generally consistent even when as much as 50% of the information came from invalid or weak instruments [19]. The MR–PRESSO method was used to detect outliers that may bias the results and to assess changes in causal estimates before and after the removal of outliers [21]. A result of *p* value < 0.05 was considered evidence of a potential association.

These sensitivity analyses were performed. An MR–Egger intercept test and an MR–PRESSO global test were used to assess horizontal pleiotropy [21]. After that, heterogeneity was tested using Cochran’s Q test in the IVW and MR–Egger methods [22]. Additionally, we conducted a leave-one-out analysis. In this analysis, we systematically removed one SNP at a time, assessing the impact of potentially pleiotropic SNPs on causal estimates. The statistical significance of the above analyses was set at a two-sided *p* value < 0.05.

For lipid-lowering agents, including instruments at moderate to low LD (r^2^ < 0.2), we applied weighted generalized linear regression for correlated IVs as described in previous studies [23]. Mendelian randomization estimated the association of statins with age at HD onset, in which statin use was equivalent to a 1 mmol/L (38.7 mg/dL) reduction in LDL cholesterol levels.

Statistical analysis was performed in R version 4.1.2 (http://cran.r-project.org/bin/windows/base/, accessed on 3 January 2022).

## 3. Results

### 3.1. Genetically Determined LDL Cholesterol and Age at Onset of HD

**Figure 1:**
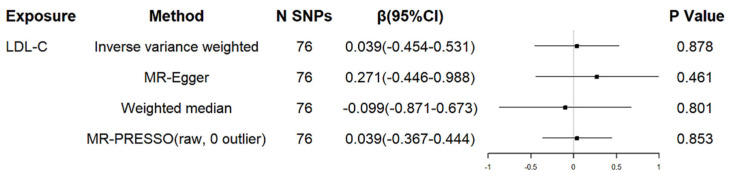
MR associations between genetically determined LDL cholesterol and residual age at onset of HD. LDL-C: low-density lipoprotein cholesterol; SNP: single nucleotide polymorphism; CI: confidence interval; HD: Huntington’s disease.

We assessed the relationship between genetically determined LDL cholesterol and the age at onset of HD. A total of 76 independent SNPs were associated with LDL cholesterol (see Supplementary Table S1). No SNPs were found to be associated with confounding factors which have been identified as having a causal relationship with AAO of HD, such as coffee consumption [24], lifetime smoking index [25], and telomere length [26]. All SNPs’ F statistics were greater than 10, which proved that the SNPs had sufficient statistical strength. All these selected 76 genetic variants could explain 1.92% variance of LDL cholesterol. The results suggested that genetically proxied LDL cholesterol was not associated with the age of HD onset (β = 0.039 years, 95% CI = −0.454 to 0.531; Figure 1) in the analysis using the IVW approach.

**Figure 2:**
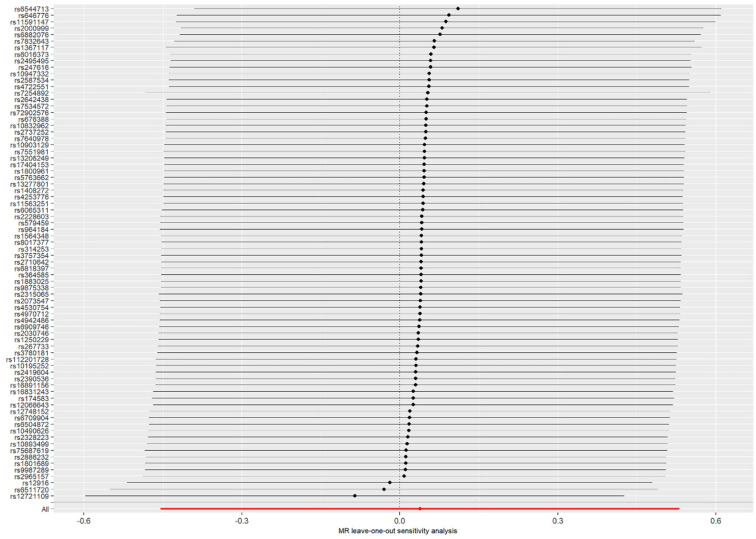
MR leave-one-out sensitivity analysis for low-density lipoprotein cholesterol on residual age at onset of HD.

The MR–Egger intercept analysis showed no evidence of horizontal pleiotropy (intercept = −0.021, *p* = 0.385) and MR–PRESSO supported that (Table 1). The causal effect estimates analyzed by MR–Egger and IVW methods suggested no evidence of heterogeneity (all *p* values > 0.05, Table 1). In the leave-one-out analysis, we did not find that a single SNP of LDL cholesterol had an influence on the association (see Figure 2).

### 3.2. Genetic Proxies for Lipid-Lowering Drugs and AAO of HD

For genetic proxies for lipid-lowering drugs, including five proxies for HMGCR (see Supplementary Table S2), no SNPs were found to be associated with confounding factors affecting AAO of HD. All SNPs had sufficient statistical strength, with F statistics greater than 10.

Genetically proxied HMG-CoA reductase inhibition equivalent to a 1 mmol/L (38.7 mg/dL) reduction in LDL cholesterol was not associated with the age at onset of HD (β = −2.228, 95% CI = −4.830 to 0.374). The heterogeneity test statistic (Cochran’s Q) was 2.8613 on 4 degrees of freedom (*p* = 0.5813).

## 4. Discussion

In this Mendelian randomization analysis of 9064 HD patients of European descent, the plasma LDL-C levels and the reduction in plasma LDL cholesterol levels by the inhibition of HMG-CoA reductase (i.e., statins) were not associated with the age of HD onset.

Alterations in cholesterol metabolism and distribution in the brain have been reported in Huntington’s disease. It is noteworthy that because of the blood–brain barrier, plasma cholesterol and brain cholesterol are two independent systems, and the increase in plasma cholesterol levels does not alter brain cholesterol levels [27]. Our study focused on the effect of peripheral blood LDL cholesterol levels on the age at onset of HD. A previous study had shown significantly decreased levels of cholesterol, HDL-C and LDL-C in HD patients [28]. In another study, plasma 24S-hydroxycholesterol (24OHC) was reduced [29]. Aziz et al. found that both HD patients and transgenic mice with a greater number of CAG replicates exhibited a higher rate of weight loss, which could be attributed to the hypermetabolic status of HD patients [30]. Larger CAG repeat sizes are indeed associated with more severe pathological conditions in the striatum and cortex [31], and may also be associated with more severe pathological status in other brain structures, such as the hypothalamus being directly involved in energy homeostasis [32]. Thus, disruption of the region involved in energy homeostasis due to the mutant protein leads to a hypermetabolic state, possibly resulting in weight loss and LDL cholesterol reduction. Weight loss or LDL cholesterol reduction in HD patients may be indications of more severe structural damage in the brain. However, whether peripheral blood LDL cholesterol is the risk factor for HD and whether it is related to the age at onset of HD still need to be further explored. In this study, we used Mendelian randomization to avoid confounding factors and reverse causality, suggesting that plasma LDL-C levels and lowering LDL-C levels by lipid-lowering agents were not associated with age of HD onset, and that LDL-C in peripheral blood may not participate in the pathogenesis of HD. This may be attributed to the fact that peripheral cholesterol cannot enter the central nervous system through the blood–brain barrier. The result might be useful in guiding the use of statins in HD patients.

In addition to its lipid-lowering effects, statins have neuroprotective effects through other mechanisms. Studies have shown that statins may have immunomodulatory effects in mice, such as decreased TNF-α, IL-1β and IL-6 levels in response to lipopolysaccharide (LPS) stimulation [33,34]. Statins have also been identified as having antioxidant properties, such as an upregulation of NO synthase, which reduces oxidative stress [10]. Simvastatin was good for the lipid balance and neuroprotection of wild-type mice and striatum-derived cells expressing mHTT, and protected against the excitatory toxicity of NMDA by decreasing the lipid content of the plasma membrane raft domain of mHTT-expressing cells [35]. In the case that statins would indeed have neuroprotective effects as suggested by previous studies, that would indicate that statin therapy should be encouraged in HD mutation carriers.

The main strengths of this analysis included the following: First, the use of genetic variants within gene-encoding drug targets to proxy the potential effects of commonly used LDL cholesterol-lowering therapies, which may minimize confounders and avoid reverse causality bias. Second, we used relatively large GWAS summary data and estimated the relationship between multiple variants in genetic proxies and outcome, allowing the increased statistical power and precision of the analysis. Moreover, there was no heterogeneity and horizontal pleiotropy in this study, which increased the robustness of the study.

This study has several limitations. First, genetics may not provide pharmacokinetic information on drug exposure. Second, given that genetic effects tend to be lifelong, we cannot distinguish critical periods of exposure to these drug targets during which disease risk may be otherwise affected. In addition, we may not be able to assess the effects of short-term use of lipid-lowering drugs. Third, valid causal inference in Mendelian randomization analysis relies on a number of assumptions, some of which are not verifiable. Although the results of this study were consistent across the various sensitivity analyses used to test the hypotheses, the possibility that these results were biased due to confounding factors and/or horizontal pleiotropy cannot be completely excluded. Finally, since the participants were of European ancestry, the results may not necessarily apply to other ethnic groups.

## 5. Conclusions

In conclusion, this study suggested that the plasma LDL-C levels and the reduction in plasma LDL cholesterol levels by the inhibition of HMG-CoA reductase (i.e., statins) were not associated with the age of HD onset. This means that statin use should not lead to the earlier onset of HD. Statins may be recommended in HD patients because other studies have shown that statins may also have neuroprotective effects, and discontinuation of statins may increase the risk of cardiovascular and cerebrovascular disease in HD patients with hyperlipidemia. MR studies combined with randomized controlled trials and other forms of pharmacoepidemiology would help to further evaluate the role of statin exposure in age at onset of HD.

## Figures and Tables

**Table 1:** Heterogeneity and pleiotropy tests of instrument effects.

Exposure	*N* SNPs	Heterogeneity Analysis				Pleiotropy Analysis			
		Method	Q	Degree of Freedom	*p*-Value	Method	Egger Intercept	SE	*p*-Value
LDL-C	76	MR Egger	50.0	74	0.985	MR Egger intercept	−0.021	0.024	0.385
		IVW	50.8	75	0.986	MR-PRESSO Global test			0.980

SNP: single nucleotide polymorphism; MR: Mendelian randomization; IVW: inverse-variance weighted; SE: standard error; LDL-C: low-density lipoprotein cholesterol.

## Data Availability

The summary statistics for LDL cholesterol are obtained from publicly available GWAS summary-level data. GeM-HD study summary statistics are freely available to qualified researchers upon request from the authors.

## Supplementary Materials

The following supporting information can be downloaded at: https://www.mdpi.com/article/10.3390/brainsci12111551/s1, Table S1: Characteristics of LDL cholesterol genetic variants; Table S2: Characteristics of genetic instruments of proxy HMG-CoA reductase.

## Abbreviations

HD: Huntington’s Disease; GWAS: genome-wide association study; MR: Mendelian randomization; IVW: inverse-variance weighted; SNP: single nucleotide polymorphism; CAG: cytosine-adenine-guanine; mHTT: mutant huntingtin; AAO: age at onset; HMG-CoA: Hydroxy-3-methylglutaryl coenzyme A; LDL: low-density lipoprotein; IVs: instrumental variables; SE: standard error; HMGCR: 3-hydroxy-3-methylglutaryl coenzyme A reductase; LPS: lipopolysaccharide.
