# Supplementary material for: Association between Genetically Proxied Inhibition of HMG-CoA Reductase and Age at Onset of Huntington’s Disease
Source: Brain Sci. 2022 Nov 15;12(11):1551. doi: 10.3390/brainsci12111551 (PMC9688104; doi:10.3390/brainsci12111551)
Supplement: Supplementary file 1 [file brainsci-12-01551-s001.zip › Table S1.pdf]

Table S1. Characteristics of LDL cholesterol genetic variants.

SNP	Effect allele	Other allele	EAF	Beta	SE	P-value	R^2	F
rs10195252	C	T	0.42	-0.024	0.0039	3.8×10^{-8}	0.000117362	37.86982249
rs10490626	A	G	0.08	-0.051	0.0069	1.7×10^{-12}	4.65E-05	54.63137996
rs10832962	C	T	0.28	-0.032	0.004	6.6×10^{-14}	0.00014923	64
rs10893499	G	A	0.86	-0.052	0.0053	3.9×10^{-21}	0.000134003	96.26201495
rs10903129	A	G	0.46	-0.033	0.0037	3.0×10^{-17}	0.000232574	79.54711468
rs10947332	G	A	0.87	-0.05	0.0056	7.0×10^{-18}	0.000106536	79.71938776
rs112201728	C	T	0.94	-0.068	0.0104	8.5×10^{-10}	5.80E-05	42.75147929
rs11563251	C	T	0.87	-0.035	0.0062	4.5×10^{-8}	4.17E-05	31.86784599
rs11591147	T	G	0.02	-0.497	0.018	8.6×10^{-143}	0.000386027	762.3734568
rs12066643	T	C	0.12	-0.039	0.0064	1.1×10^{-8}	4.58E-05	37.13378906
rs1250229	T	C	0.21	-0.024	0.0042	3.1×10^{-8}	6.26E-05	32.65306122
rs12721109	A	G	0.02	-0.446	0.0183	3.0×10^{-122}	0.000234222	593.9741408
rs12748152	C	T	0.93	-0.05	0.0066	3.2×10^{-12}	4.32E-05	57.39210285
rs12916	T	C	0.57	-0.073	0.0038	7.8×10^{-78}	0.001074535	369.0443213
rs13206249	A	G	0.22	-0.038	0.0062	4.5×10^{-08}	0.000147934	37.56503642
rs13277801	T	C	0.65	-0.034	0.0038	4.0×10^{-17}	0.000210538	80.05540166
rs1367117	G	A	0.71	-0.119	0.004	9.5×10^{-183}	0.00210667	885.0625
rs1408272	G	T	0.05	-0.052	0.0083	3.7×10^{-09}	2.22E-05	39.25097982
rs1564348	T	C	0.85	-0.048	0.005	2.8×10^{-21}	0.000135851	92.16
rs16831243	C	T	0.82	-0.038	0.0055	9.1×10^{-12}	8.65E-05	47.73553719
rs16891156	A	C	0.98	-0.097	0.0171	8.2×10^{-09}	1.39E-05	32.17742211
rs17404153	T	G	0.14	-0.034	0.0054	1.8×10^{-09}	5.52E-05	39.64334705
rs174583	T	C	0.37	-0.052	0.0038	7.0×10^{-41}	0.000504674	187.2576177
rs1800961	T	C	0.03	-0.069	0.0106	6.0×10^{-10}	1.73E-05	42.37273051
rs1801689	A	C	0.96	-0.103	0.0139	9.8×10^{-12}	3.79E-05	54.90916619
rs1883025	T	C	0.24	-0.03	0.0044	6.1×10^{-11}	9.84E-05	46.48760331
rs2000999	G	A	0.82	-0.065	0.0046	4.2×10^{-41}	0.000343667	199.6691871
rs2030746	C	T	0.6	-0.021	0.0038	8.6×10^{-09}	8.47E-05	30.5401662
rs2073547	A	G	0.81	-0.049	0.0049	1.9×10^{-21}	0.000181177	100
rs2228603	T	C	0.07	-0.104	0.0072	4.4×10^{-44}	0.000171235	208.6419753
rs2315065	C	A	0.91	-0.11	0.0158	5.2×10^{-12}	0.000117714	48.46979651
rs2328223	A	C	0.75	-0.03	0.005	5.6×10^{-09}	7.91E-05	36
rs2390536	G	A	0.63	-0.022	0.0038	2.0×10^{-08}	9.03E-05	33.51800554
rs2419604	G	A	0.68	-0.03	0.004	7.5×10^{-14}	0.000141661	56.25
rs247616	T	C	0.29	-0.055	0.0041	2.6×10^{-37}	0.000432201	179.9524093
rs2495495	C	T	0.87	-0.034	0.0059	3.5×10^{-08}	4.63E-05	33.20884803
rs2587534	G	A	0.47	-0.039	0.0037	8.1×10^{-25}	0.000320014	111.1029949
rs2642438	A	G	0.25	-0.035	0.0042	7.3×10^{-16}	0.00015738	69.44444444
rs267733	G	A	0.14	-0.033	0.0053	5.3×10^{-09}	5.67E-05	38.76824493
rs2710642	G	A	0.38	-0.024	0.0038	6.1×10^{-09}	0.00010865	39.88919668

rs2737252	A	G	0.26	-0.031	0.0041	7.0×10^{-14}	0.000127195	57.16835217
rs2886232	C	T	0.88	-0.045	0.0064	3.9×10^{-11}	6.43E-05	49.43847656
rs2965157	C	T	0.02	-0.189	0.0112	7.3×10^{-62}	6.56E-05	284.765625
rs314253	C	T	0.34	-0.024	0.0038	3.4×10^{-10}	0.00010549	39.88919668
rs364585	A	G	0.37	-0.025	0.0038	4.3×10^{-10}	0.00011764	43.28254848
rs3757354	T	C	0.21	-0.038	0.0044	2.1×10^{-17}	0.000143062	74.58677686
rs3780181	G	A	0.05	-0.045	0.0074	1.8×10^{-09}	2.04E-05	36.97954711
rs4253776	A	G	0.88	-0.031	0.0059	3.4×10^{-08}	3.41E-05	27.60700948
rs4530754	G	A	0.42	-0.028	0.0036	3.6×10^{-12}	0.000170359	60.49382716
rs4722551	T	C	0.83	-0.039	0.0049	4.0×10^{-14}	0.000103367	63.34860475
rs4942486	C	T	0.54	-0.024	0.0037	2.3×10^{-11}	0.000121576	42.07450694
rs4970712	A	C	0.19	-0.034	0.0044	2.5×10^{-13}	0.000106215	59.7107438
rs5763662	C	T	0.97	-0.077	0.0121	1.2×10^{-08}	1.45E-05	40.49586777
rs579459	T	C	0.79	-0.067	0.0045	2.4×10^{-44}	0.000425886	221.6790123
rs6016373	G	A	0.37	-0.035	0.0037	8.0×10^{-19}	0.00024316	89.48137327
rs6065311	T	C	0.54	-0.042	0.0036	1.7×10^{-30}	0.00039467	136.1111111
rs646776	C	T	0.21	-0.16	0.0044	1.6×10^{-272}	0.002535784	1322.31405
rs6504872	C	T	0.53	-0.027	0.0037	3.5×10^{-13}	0.000154673	53.25054785
rs6511720	T	G	0.1	-0.221	0.0061	3.9×10^{-262}	0.001384835	1312.577264
rs6544713	C	T	0.71	-0.081	0.0041	4.8×10^{-83}	0.00092938	390.3033908
rs6709904	G	A	0.11	-0.055	0.0085	4.6×10^{-10}	9.12E-05	41.86851211
rs676388	T	C	0.54	-0.027	0.0039	1.3×10^{-11}	0.000142727	47.92899408
rs6818397	G	T	0.59	-0.022	0.004	1.7×10^{-08}	8.47E-05	30.25
rs6882076	T	C	0.33	-0.046	0.0038	3.3×10^{-31}	0.000374547	146.5373961
rs6909746	T	C	0.39	-0.026	0.0037	7.9×10^{-11}	0.000138125	49.37910884
rs7254892	A	G	0.03	-0.485	0.0119	3.85×10^{-326}	0.000694512	1661.076195
rs72902576	G	T	0.04	-0.093	0.0133	9.6×10^{-12}	4.58E-05	48.89479337
rs7534572	C	G	0.31	-0.041	0.0058	1.3×10^{-11}	0.00028448	49.97027348
rs7551981	G	T	0.41	-0.047	0.0038	1.4×10^{-33}	0.000427755	152.9778393
rs75687619	G	T	0.98	-0.174	0.0161	8.1×10^{-24}	5.58E-05	116.8010493
rs7640978	T	C	0.11	-0.039	0.0069	9.8×10^{-09}	3.63E-05	31.94706994
rs7832643	G	T	0.6	-0.034	0.0038	2.7×10^{-17}	0.000233095	80.05540166
rs8017377	G	A	0.54	-0.03	0.0038	2.5×10^{-15}	0.000179121	62.32686981
rs964184	C	G	0.84	-0.086	0.0078	2.0×10^{-26}	0.000363615	121.56476
rs9875338	A	G	0.39	-0.027	0.0037	2.2×10^{-11}	0.000146543	53.25054785
rs9987289	A	G	0.08	-0.071	0.0066	8.5×10^{-24}	0.0001064	115.7254362

SNP: single nucleotide polymorphism; EAF: effect allele frequency; SE: standard error.
