# Supplementary material for: Association between Genetically Proxied Inhibition of HMG-CoA Reductase and Age at Onset of Huntington’s Disease
Source: Brain Sci. 2022 Nov 15;12(11):1551. doi: 10.3390/brainsci12111551 (PMC9688104; doi:10.3390/brainsci12111551)
Supplement: Supplementary file 1 [file brainsci-12-01551-s001.zip › Table S2.pdf]

Table S2. Characteristics of genetic instruments of proxy HMG-CoA reductase.

SNP	Effect allele	Other allele	EAF	Beta	SE	P-value	R^2	F
**HMG-CoA reductase**								
rs10515198	G	A	0.9	-0.06	0.0061	6.0×10^{-22}	0.000100648	96.74818597
rs12173076	T	G	0.88	-0.065	0.0061	2.3×10^{-27}	0.000142439	113.544746
rs12916	T	C	0.57	-0.073	0.0041	7.8×10^{-78}	0.000898204	317.0136823
rs3857388	T	C	0.87	-0.042	0.0061	2.2×10^{-11}	6.20E-05	47.40661113
rs7711235	A	G	0.73	-0.038	0.0061	5.0×10^{-10}	0.000170497	38.80677237

SNP: single nucleotide polymorphism; EAF: effect allele frequency; SE: standard error;

HMG-CoA: 3-hydroxy-3-methylglutaryl coenzyme A.
